# (2*E*)-2-[1-(2-Hy­droxy-4-meth­oxy­phenyl)ethyl­idene]-*N*-phenyl­hydrazine­carbox­amide monohydrate

**DOI:** 10.1107/S1600536812039414

**Published:** 2012-09-22

**Authors:** C. F. Annie, Jinsa Mary Jacob, M. Sithambaresan, M. R. Prathapachandra Kurup

**Affiliations:** a Department of Applied Chemistry, Cochin University of Science and Technology, Kochi 682 022, India; b Department of Chemistry, Faculty of Science, Eastern University, Sri Lanka, Chenkalady, Sri Lanka

## Abstract

The title compound, C_16_H_17_N_3_O_3_·H_2_O, exists in the *E* conformation with respect to the azomethine C=N double bond. While the phenyl ring is almost coplanar with the central hydrazinecarboxamide group [dihedral angle = 14.18 (11)°], it is twisted slightly with respect to the other aromatic ring in the mol­ecule, with a dihedral angle of 22.88 (13)°. The packing is dominated by O—H⋯O, N—H⋯O and C—H⋯O hydrogen-bond inter­actions, forming a three-dimensional supra­molecular structure which is augmented by two types of C—H⋯π inter­actions. An intramolecular O—H⋯N interaction is also present in the molecule.

## Related literature

For the application of hydrazinecarboxamides as enzyme inhibitors and as a source of self-complementary bidirectional hydrogen-bonding motifs, see: Lam *et al.* (1994 ▶); Chorev & Goodman (1993 ▶); Zhao *et al.* (1990 ▶). For the synthesis of related compounds, see: Sreekanth *et al.* (2004 ▶). For standard bond-length data, see: Allen *et al.* (1987 ▶). For related structures, see: Sithambaresan & Kurup (2011 ▶); Siji *et al.* (2010 ▶).
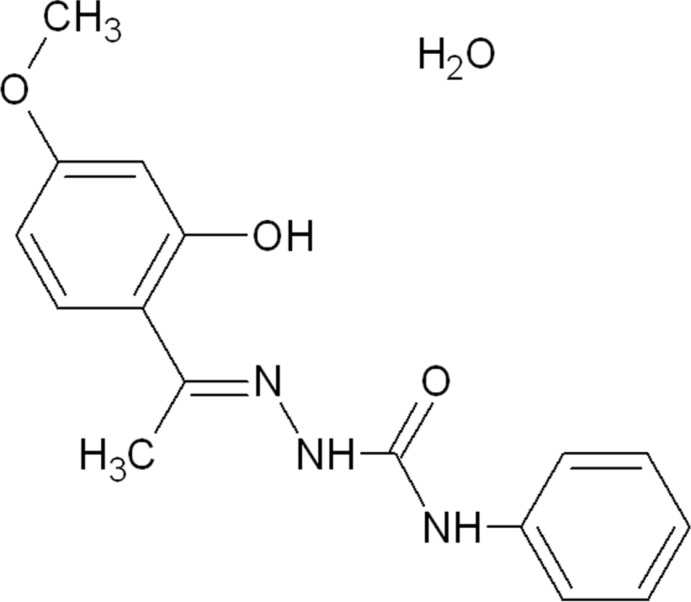


## Experimental

### 

#### Crystal data

C_16_H_17_N_3_O_3_·H_2_O
*M* *_r_* = 317.34
Monoclinic, 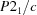

*a* = 12.4020 (18) Å
*b* = 13.7808 (19) Å
*c* = 9.3919 (10) Å
β = 96.813 (7)°
*V* = 1593.8 (4) Å^3^
*Z* = 4
Mo *K*α radiation
μ = 0.10 mm^−1^
*T* = 296 K
0.50 × 0.30 × 0.25 mm

#### Data collection

Bruker Kappa APEXII CCD diffractometer
Absorption correction: multi-scan (*SADABS* (Bruker, 2004 ▶) *T*_min_ = 0.966, *T*_max_ = 0.976
12097 measured reflections
2809 independent reflections
1807 reflections with *I* > 2σ(*I*)
*R*_int_ = 0.083

#### Refinement

*R*[*F* ^2^ > 2σ(*F* ^2^)] = 0.053
*wR*(*F* ^2^) = 0.172
*S* = 1.02
2809 reflections
230 parameters
6 restraints
H atoms treated by a mixture of independent and constrained refinement
Δρ_max_ = 0.22 e Å^−3^
Δρ_min_ = −0.19 e Å^−3^

### 

Data collection: *APEX2* (Bruker, 2004 ▶); cell refinement: *APEX2* and *SAINT* (Bruker, 2004 ▶); data reduction: *SAINT* and *XPREP* (Bruker, 2004 ▶); program(s) used to solve structure: *SHELXL97* (Sheldrick, 2008 ▶); program(s) used to refine structure: *SHELXL97* (Sheldrick, 2008 ▶); molecular graphics: *ORTEP-3* (Farrugia, 1997 ▶) and *DIAMOND* (Brandenburg, 2010 ▶); software used to prepare material for publication: *SHELXL97* and *publCIF* (Westrip, 2010 ▶).

## Supplementary Material

Crystal structure: contains datablock(s) I, global. DOI: 10.1107/S1600536812039414/bv2210sup1.cif

Structure factors: contains datablock(s) I. DOI: 10.1107/S1600536812039414/bv2210Isup2.hkl

Supplementary material file. DOI: 10.1107/S1600536812039414/bv2210Isup3.cml

Additional supplementary materials:  crystallographic information; 3D view; checkCIF report

## Figures and Tables

**Table 1:** Hydrogen-bond geometry (Å, °) *Cg*1 is the centroid of the C1–C6 ring.

*D*—H⋯*A*	*D*—H	H⋯*A*	*D*⋯*A*	*D*—H⋯*A*
O2—H2′⋯N1	0.88 (1)	1.71 (2)	2.526 (2)	154 (3)
N2—H2N⋯O1*S* ^i^	0.88 (1)	2.08 (1)	2.900 (3)	154 (2)
N3—H3⋯O1*S* ^i^	0.88 (1)	2.11 (2)	2.918 (3)	153 (3)
O1*S*—H1*A*⋯O2	0.86 (2)	2.12 (2)	2.925 (3)	156 (3)
O1*S*—H1*B*⋯O3^ii^	0.84 (2)	1.90 (2)	2.730 (3)	174 (3)
C8—H8*C*⋯O3^iii^	0.96	2.51	3.457 (3)	167
C11—H11⋯O3	0.93	2.31	2.881 (3)	119
C13—H13⋯O1^iv^	0.93	2.60	3.489 (3)	160
C8—H8*A*⋯*Cg*1^v^	0.96	2.92	3.543 (3)	123
C16—H16*C*⋯*Cg*1^vi^	0.96	2.79	3.645 (4)	148

Symmetry codes: (i) 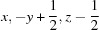; (ii) 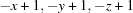; (iii) 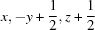; (iv) 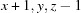; (v) 
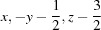
; (vi) 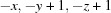.

**Fig. 1.**
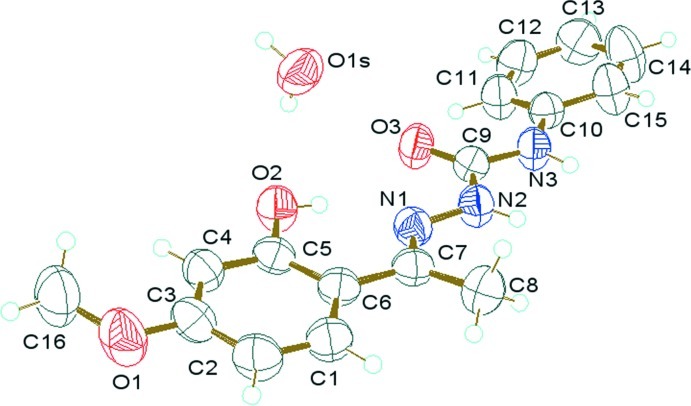
ORTEP view of the compound, drawn with 50% probability displacement ellipsoids for the non-H atoms.

**Fig. 2.**
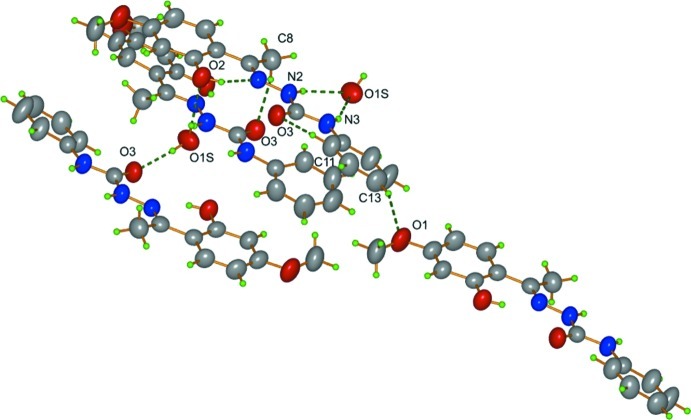
Graphical representation showing three-dimensional supramolecular hydrogen bonding network in the crystal structure of C16H17N3O3.H2O.

**Fig. 3.**
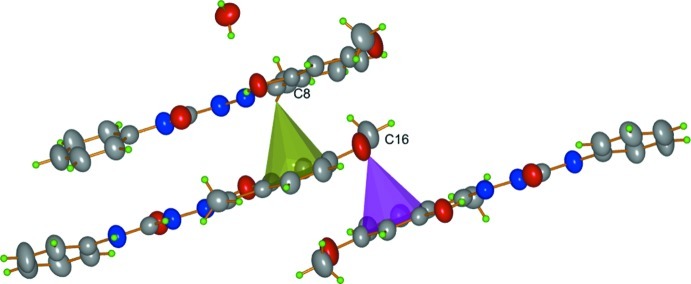
C—H···π interactions present in the compound C16H17N3O3.H2O.

**Fig. 4.**
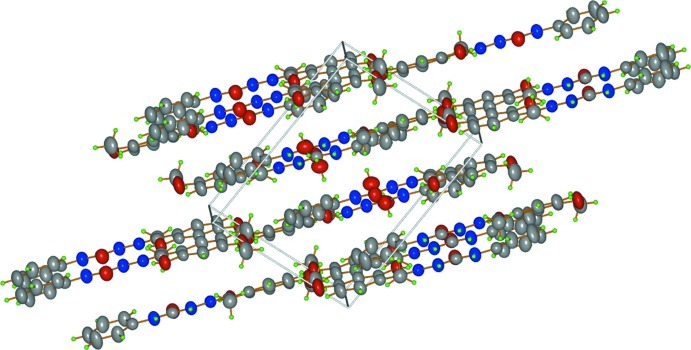
Packing diagram of the compound showing the parallel arrangement of the molecules along b axis.

## supplementary crystallographic information

### Comment

Hydrazinecarboxamides have gained considerable importance in recent years in the design of enzyme inhibitors (Lam *et al.*, 1994), as replacement for the amide (–CO–NH–) bond in peptidomimetics (Chorev & Goodman, 1993) and as sources of self-complementary bidirectional hydrogen bonding motif in supramolecular chemistry (Zhao *et al.*, 1990). As a continuous work on the hydrazinecarboxamide compounds, a new hydrazinecarboxamide compound, (2*E*)-2-[1-(2-hydroxy-4-methoxyphenyl)ethylidene]-*N*-phenylhydrazinecarboxamide monohydrate, was prepared and structurally characterized. The *ORTEP* view of the title compound is shown in Fig. 1.

The compound crystallizes in monoclinic space group *P*2_1_/c. The molecule adopts an *E* configuration with respect to C7═N1 bond (Sithambaresan & Kurup, 2011; Siji *et al.*, 2010) and it exists in amido form with C9═O3 bond length of 1.212 (3) Å which is very close to a formal C═O bond length [1.21 Å] (Allen *et al.*, 1987). The phenyl ring is almost coplanar with the central hydrazinecarboxamide moiety with maximum deviation of -0.060 (3) Å for the C1 atom. The two aromatic rings are twisted with dihedral angle of 22.88 (13)°.

While the intramolecular O—H···N and C—H···O hydrogen bonds increase the rigidity of the molecule, intermolecular O—H···O, N—H···O, C—H···O hydrogen bonding interactions (Table 1) links the adjacent molecules directly and through water molecule forming an infinite three-dimensional supramolecular structure in the lattice (Fig. 2). Phenylhydrazinecarboxamide molecules also interact through two types of C—H···π interactions (Fig. 3) with the H···π distances of 2.92 and 2.79 Å and very weak π–π interactions with a shortest centroid–centroid distance of 5.0552 (18) Å. The parallel arrangement of the molecules along *b* axis is shown in Fig. 4.

### Experimental

The title compound was prepared by adapting a reported procedure (Sreekanth *et al.*, 2004). To a warm ethanolic solution of *N*-phenylsemicarbazide (0.302 g, 2 mmol), an ethanolic solution of 1-(2-hydroxy-4-methoxyphenyl)ethanone (0.332 g, 2 mmol) was added and the resulting solution was refluxed for 3 h after adding three drops of glacial acetic acid. On cooling the solution colorless crystals were separated out. Single crystals suitable for X-ray diffraction studies were obtained by slow evaporation from its ethanolic solution.

### Refinement

All H atoms on C were placed in calculated positions, guided by difference maps, with C—H bond distances 0.93–0.96 Å. H atoms were assigned as *U*_iso_ = 1.2Ueq. H1A and H1B atoms of O1S were located from difference maps and restrained using DFIX and DANG instructions with O—H = 0.86 (2) and H···H = 1.36 (2) Å respectively. N2—H2N, N3—H3 and O2—H2' atoms were located from difference maps and restrained using DFIX instructions with bond distance of 0.88 (1) Å.

### Crystal data

**Table:**

C_16_H_17_N_3_O_3_·H_2_O	*F*(000) = 672
*M**_r_* = 317.34	*D*_x_ = 1.322 Mg m^−^^3^
Monoclinic, *P*2_1_/*c*	Mo *K*α radiation, λ = 0.71073 Å
Hall symbol: -P 2ybc	Cell parameters from 2335 reflections
*a* = 12.4020 (18) Å	θ = 2.6–28.5°
*b* = 13.7808 (19) Å	µ = 0.10 mm^−^^1^
*c* = 9.3919 (10) Å	*T* = 296 K
β = 96.813 (7)°	Block, light yellow
*V* = 1593.8 (4) Å^3^	0.50 × 0.30 × 0.25 mm
*Z* = 4	

### Data collection

**Table:**

Bruker Kappa APEXII CCD diffractometer	2809 independent reflections
Radiation source: fine-focus sealed tube	1807 reflections with *I* > 2σ(*I*)
Graphite monochromator	*R*_int_ = 0.083
Detector resolution: 8.33 pixels mm^-1^	θ_max_ = 25.0°, θ_min_ = 2.6°
ω and φ scans	*h* = −14→14
Absorption correction: multi-scan (*SADABS* (Bruker, 2004)	*k* = −15→16
*T*_min_ = 0.966, *T*_max_ = 0.976	*l* = −11→11
12097 measured reflections	

### Refinement

**Table:**

Refinement on *F*^2^	Primary atom site location: structure-invariant direct methods
Least-squares matrix: full	Secondary atom site location: difference Fourier map
*R*[*F*^2^ > 2σ(*F*^2^)] = 0.053	Hydrogen site location: inferred from neighbouring sites
*wR*(*F*^2^) = 0.172	H atoms treated by a mixture of independent and constrained refinement
*S* = 1.02	*w* = 1/[σ^2^(*F*_o_^2^) + (0.0711*P*)^2^ + 0.2072*P*] where *P* = (*F*_o_^2^ + 2*F*_c_^2^)/3
2809 reflections	(Δ/σ)_max_ = 0.002
230 parameters	Δρ_max_ = 0.22 e Å^−^^3^
6 restraints	Δρ_min_ = −0.19 e Å^−^^3^

### Special details

**Table:**

Geometry. All e.s.d.'s (except the e.s.d. in the dihedral angle between two l.s. planes) are estimated using the full covariance matrix. The cell e.s.d.'s are taken into account individually in the estimation of e.s.d.'s in distances, angles and torsion angles; correlations between e.s.d.'s in cell parameters are only used when they are defined by crystal symmetry. An approximate (isotropic) treatment of cell e.s.d.'s is used for estimating e.s.d.'s involving l.s. planes.
Refinement. Refinement of *F*^2^ against ALL reflections. The weighted *R*-factor *wR* and goodness of fit *S* are based on *F*^2^, conventional *R*-factors *R* are based on *F*, with *F* set to zero for negative *F*^2^. The threshold expression of *F*^2^ > σ(*F*^2^) is used only for calculating *R*-factors(gt) *etc*. and is not relevant to the choice of reflections for refinement. *R*-factors based on *F*^2^ are statistically about twice as large as those based on *F*, and *R*-factors based on ALL data will be even larger.

### Fractional atomic coordinates and isotropic or equivalent isotropic displacement parameters (Å^2^)

**Table:**

	*x*	*y*	*z*	*U*_iso_*/*U*_eq_	
O1	0.00919 (16)	0.45528 (15)	0.7326 (2)	0.0873 (6)	
O2	0.28586 (14)	0.46101 (12)	0.42646 (18)	0.0649 (5)	
O3	0.48111 (13)	0.39835 (12)	0.18956 (17)	0.0673 (5)	
O1S	0.46607 (17)	0.43758 (13)	0.6547 (2)	0.0782 (6)	
N1	0.34225 (14)	0.30092 (14)	0.32720 (19)	0.0550 (5)	
N2	0.41511 (16)	0.25435 (15)	0.2542 (2)	0.0579 (5)	
N3	0.55055 (16)	0.25697 (15)	0.1149 (2)	0.0595 (5)	
C1	0.1243 (2)	0.26313 (19)	0.5438 (3)	0.0665 (7)	
H1	0.1149	0.1964	0.5341	0.080*	
C2	0.0592 (2)	0.3136 (2)	0.6243 (3)	0.0721 (7)	
H2	0.0057	0.2814	0.6674	0.087*	
C3	0.07267 (18)	0.41231 (19)	0.6422 (3)	0.0632 (6)	
C4	0.14813 (18)	0.46014 (18)	0.5735 (2)	0.0605 (6)	
H4	0.1560	0.5270	0.5831	0.073*	
C5	0.21263 (16)	0.40877 (17)	0.4898 (2)	0.0525 (6)	
C6	0.20481 (17)	0.30831 (17)	0.4753 (2)	0.0527 (6)	
C7	0.27824 (18)	0.25124 (17)	0.3976 (2)	0.0549 (6)	
C8	0.2817 (2)	0.14371 (19)	0.4067 (3)	0.0725 (7)	
H8A	0.2911	0.1172	0.3145	0.109*	
H8B	0.2149	0.1202	0.4363	0.109*	
H8C	0.3413	0.1242	0.4754	0.109*	
C9	0.48365 (17)	0.31043 (17)	0.1865 (2)	0.0528 (5)	
C10	0.63021 (18)	0.29082 (16)	0.0333 (2)	0.0544 (6)	
C11	0.6348 (2)	0.38417 (19)	−0.0166 (3)	0.0703 (7)	
H11	0.5839	0.4297	0.0053	0.084*	
C12	0.7142 (2)	0.4102 (2)	−0.0987 (3)	0.0797 (8)	
H12	0.7167	0.4735	−0.1323	0.096*	
C13	0.7897 (2)	0.3447 (2)	−0.1319 (3)	0.0858 (9)	
H13	0.8439	0.3628	−0.1870	0.103*	
C14	0.7843 (3)	0.2526 (3)	−0.0828 (4)	0.0974 (11)	
H14	0.8355	0.2074	−0.1046	0.117*	
C15	0.7047 (2)	0.2249 (2)	−0.0017 (3)	0.0787 (8)	
H15	0.7016	0.1611	0.0295	0.094*	
C16	0.0259 (3)	0.5537 (2)	0.7664 (3)	0.0955 (10)	
H16A	0.1001	0.5636	0.8055	0.143*	
H16B	−0.0211	0.5728	0.8357	0.143*	
H16C	0.0100	0.5920	0.6812	0.143*	
H1A	0.424 (3)	0.459 (3)	0.583 (3)	0.141 (16)*	
H1B	0.486 (3)	0.4887 (17)	0.698 (3)	0.109 (12)*	
H3	0.548 (2)	0.1938 (8)	0.126 (3)	0.089 (9)*	
H2'	0.318 (2)	0.4169 (16)	0.378 (3)	0.094 (10)*	
H2N	0.4174 (19)	0.1907 (8)	0.245 (2)	0.063 (7)*	

### Atomic displacement parameters (Å^2^)

**Table:**

	*U*^11^	*U*^22^	*U*^33^	*U*^12^	*U*^13^	*U*^23^
O1	0.0817 (13)	0.0877 (15)	0.1018 (14)	0.0039 (10)	0.0499 (10)	0.0000 (11)
O2	0.0673 (11)	0.0548 (11)	0.0776 (11)	−0.0040 (8)	0.0296 (8)	0.0036 (8)
O3	0.0756 (11)	0.0487 (10)	0.0802 (12)	0.0015 (8)	0.0203 (8)	−0.0082 (8)
O1S	0.0866 (13)	0.0528 (11)	0.0914 (14)	−0.0062 (10)	−0.0048 (11)	−0.0055 (10)
N1	0.0542 (11)	0.0577 (13)	0.0540 (11)	0.0038 (9)	0.0097 (8)	−0.0022 (8)
N2	0.0619 (12)	0.0503 (13)	0.0637 (12)	0.0046 (9)	0.0178 (9)	−0.0037 (9)
N3	0.0641 (12)	0.0469 (12)	0.0710 (13)	0.0047 (9)	0.0225 (10)	−0.0005 (9)
C1	0.0627 (15)	0.0601 (16)	0.0788 (17)	−0.0095 (12)	0.0176 (13)	0.0023 (12)
C2	0.0637 (15)	0.0726 (19)	0.0847 (18)	−0.0111 (13)	0.0283 (13)	0.0069 (14)
C3	0.0536 (14)	0.0717 (18)	0.0668 (15)	0.0032 (12)	0.0176 (11)	0.0055 (12)
C4	0.0558 (13)	0.0601 (15)	0.0673 (15)	0.0026 (11)	0.0138 (11)	0.0012 (11)
C5	0.0459 (12)	0.0585 (15)	0.0536 (13)	−0.0029 (10)	0.0077 (9)	0.0074 (10)
C6	0.0494 (12)	0.0576 (15)	0.0505 (12)	−0.0026 (10)	0.0037 (9)	0.0044 (10)
C7	0.0519 (13)	0.0595 (15)	0.0518 (13)	−0.0010 (10)	−0.0001 (10)	0.0014 (10)
C8	0.0812 (18)	0.0603 (16)	0.0766 (17)	−0.0002 (13)	0.0116 (13)	−0.0021 (12)
C9	0.0521 (13)	0.0493 (14)	0.0560 (14)	0.0013 (10)	0.0023 (10)	−0.0033 (10)
C10	0.0536 (13)	0.0552 (15)	0.0548 (13)	0.0017 (10)	0.0076 (10)	−0.0023 (10)
C11	0.0765 (17)	0.0573 (16)	0.0808 (17)	0.0057 (13)	0.0251 (13)	0.0006 (13)
C12	0.097 (2)	0.0648 (18)	0.0831 (19)	−0.0106 (15)	0.0328 (15)	0.0019 (13)
C13	0.0797 (19)	0.095 (2)	0.089 (2)	−0.0092 (16)	0.0368 (15)	−0.0005 (17)
C14	0.093 (2)	0.089 (2)	0.120 (3)	0.0294 (17)	0.0550 (19)	0.0167 (18)
C15	0.0864 (19)	0.0643 (18)	0.091 (2)	0.0191 (14)	0.0335 (15)	0.0115 (14)
C16	0.100 (2)	0.091 (3)	0.103 (2)	0.0114 (18)	0.0410 (18)	−0.0163 (18)

### Geometric parameters (Å, º)

**Table:**

O1—C3	1.360 (3)	C4—H4	0.9300
O1—C16	1.403 (3)	C5—C6	1.393 (3)
O2—C5	1.351 (3)	C6—C7	1.462 (3)
O2—H2'	0.881 (10)	C7—C8	1.485 (3)
O3—C9	1.212 (3)	C8—H8A	0.9600
O1S—H1A	0.857 (18)	C8—H8B	0.9600
O1S—H1B	0.837 (18)	C8—H8C	0.9600
N1—C7	1.289 (3)	C10—C15	1.363 (3)
N1—N2	1.358 (3)	C10—C11	1.373 (3)
N2—C9	1.361 (3)	C11—C12	1.368 (3)
N2—H2N	0.882 (10)	C11—H11	0.9300
N3—C9	1.348 (3)	C12—C13	1.363 (4)
N3—C10	1.400 (3)	C12—H12	0.9300
N3—H3	0.878 (10)	C13—C14	1.354 (4)
C1—C2	1.360 (4)	C13—H13	0.9300
C1—C6	1.397 (3)	C14—C15	1.370 (4)
C1—H1	0.9300	C14—H14	0.9300
C2—C3	1.379 (4)	C15—H15	0.9300
C2—H2	0.9300	C16—H16A	0.9600
C3—C4	1.368 (3)	C16—H16B	0.9600
C4—C5	1.381 (3)	C16—H16C	0.9600
			
C3—O1—C16	118.8 (2)	C7—C8—H8B	109.5
C5—O2—H2'	103 (2)	H8A—C8—H8B	109.5
H1A—O1S—H1B	102 (3)	C7—C8—H8C	109.5
C7—N1—N2	119.7 (2)	H8A—C8—H8C	109.5
N1—N2—C9	117.21 (19)	H8B—C8—H8C	109.5
N1—N2—H2N	123.3 (16)	O3—C9—N3	125.3 (2)
C9—N2—H2N	119.4 (16)	O3—C9—N2	122.5 (2)
C9—N3—C10	127.4 (2)	N3—C9—N2	112.2 (2)
C9—N3—H3	117 (2)	C15—C10—C11	119.0 (2)
C10—N3—H3	115 (2)	C15—C10—N3	116.9 (2)
C2—C1—C6	122.1 (2)	C11—C10—N3	124.0 (2)
C2—C1—H1	118.9	C12—C11—C10	120.0 (2)
C6—C1—H1	118.9	C12—C11—H11	120.0
C1—C2—C3	120.0 (2)	C10—C11—H11	120.0
C1—C2—H2	120.0	C13—C12—C11	121.0 (3)
C3—C2—H2	120.0	C13—C12—H12	119.5
O1—C3—C4	124.3 (2)	C11—C12—H12	119.5
O1—C3—C2	115.8 (2)	C14—C13—C12	118.6 (3)
C4—C3—C2	119.9 (2)	C14—C13—H13	120.7
C3—C4—C5	119.8 (2)	C12—C13—H13	120.7
C3—C4—H4	120.1	C13—C14—C15	121.2 (3)
C5—C4—H4	120.1	C13—C14—H14	119.4
O2—C5—C4	116.3 (2)	C15—C14—H14	119.4
O2—C5—C6	121.9 (2)	C10—C15—C14	120.1 (3)
C4—C5—C6	121.8 (2)	C10—C15—H15	119.9
C5—C6—C1	116.3 (2)	C14—C15—H15	119.9
C5—C6—C7	122.9 (2)	O1—C16—H16A	109.5
C1—C6—C7	120.8 (2)	O1—C16—H16B	109.5
N1—C7—C6	115.4 (2)	H16A—C16—H16B	109.5
N1—C7—C8	123.0 (2)	O1—C16—H16C	109.5
C6—C7—C8	121.6 (2)	H16A—C16—H16C	109.5
C7—C8—H8A	109.5	H16B—C16—H16C	109.5
			
C7—N1—N2—C9	−177.41 (18)	C5—C6—C7—N1	−8.6 (3)
C6—C1—C2—C3	−1.0 (4)	C1—C6—C7—N1	173.59 (19)
C16—O1—C3—C4	−4.5 (4)	C5—C6—C7—C8	168.3 (2)
C16—O1—C3—C2	174.0 (2)	C1—C6—C7—C8	−9.5 (3)
C1—C2—C3—O1	−175.5 (2)	C10—N3—C9—O3	1.1 (4)
C1—C2—C3—C4	3.0 (4)	C10—N3—C9—N2	179.5 (2)
O1—C3—C4—C5	176.5 (2)	N1—N2—C9—O3	−0.1 (3)
C2—C3—C4—C5	−1.8 (4)	N1—N2—C9—N3	−178.56 (18)
C3—C4—C5—O2	−179.4 (2)	C9—N3—C10—C15	165.1 (2)
C3—C4—C5—C6	−1.4 (3)	C9—N3—C10—C11	−17.3 (4)
O2—C5—C6—C1	−178.8 (2)	C15—C10—C11—C12	−0.8 (4)
C4—C5—C6—C1	3.3 (3)	N3—C10—C11—C12	−178.4 (2)
O2—C5—C6—C7	3.4 (3)	C10—C11—C12—C13	−0.2 (4)
C4—C5—C6—C7	−174.6 (2)	C11—C12—C13—C14	0.5 (5)
C2—C1—C6—C5	−2.1 (3)	C12—C13—C14—C15	0.1 (5)
C2—C1—C6—C7	175.8 (2)	C11—C10—C15—C14	1.5 (4)
N2—N1—C7—C6	178.56 (17)	N3—C10—C15—C14	179.2 (3)
N2—N1—C7—C8	1.7 (3)	C13—C14—C15—C10	−1.2 (5)

### Hydrogen-bond geometry (Å, º)

Cg1 is the centroid of the C1–C6 ring.

**Table:**

*D*—H···*A*	*D*—H	H···*A*	*D*···*A*	*D*—H···*A*
O2—H2′···N1	0.88 (1)	1.71 (2)	2.526 (2)	154 (3)
N2—H2*N*···O1*S*^i^	0.88 (1)	2.08 (1)	2.900 (3)	154 (2)
N3—H3···O1*S*^i^	0.88 (1)	2.11 (2)	2.918 (3)	153 (3)
O1*S*—H1*A*···O2	0.86 (2)	2.12 (2)	2.925 (3)	156 (3)
O1*S*—H1*B*···O3^ii^	0.84 (2)	1.90 (2)	2.730 (3)	174 (3)
C8—H8*C*···O3^iii^	0.96	2.51	3.457 (3)	167
C11—H11···O3	0.93	2.31	2.881 (3)	119
C13—H13···O1^iv^	0.93	2.60	3.489 (3)	160
C8—H8*A*···*Cg*1^v^	0.96	2.92	3.543 (3)	123
C16—H16*C*···*Cg*1^vi^	0.96	2.79	3.645 (4)	148

Symmetry codes: (i) *x*, −*y*+1/2, *z*−1/2; (ii) −*x*+1, −*y*+1, −*z*+1; (iii) *x*, −*y*+1/2, *z*+1/2; (iv) *x*+1, *y*, *z*−1; (v) *x*, −*y*−1/2, *z*−3/2; (vi) −*x*, −*y*+1, −*z*+1.
